# HIV-2 mediated effects on target and bystander cells induce plasma proteome remodeling

**DOI:** 10.1016/j.isci.2024.109344

**Published:** 2024-02-28

**Authors:** Emil Johansson, Jamirah Nazziwa, Eva Freyhult, Mun-Gwan Hong, Jacob Lindman, Malin Neptin, Sara Karlson, Melinda Rezeli, Antonio J. Biague, Patrik Medstrand, Fredrik Månsson, Hans Norrgren, Joakim Esbjörnsson, Marianne Jansson, for the SWEGUB CORE group

**Affiliations:** 1 Department of Translational Medicine, Lund University, Lund, Sweden; 2 Lund University Virus Centre, Lund, Sweden; 3 Department of Cell and Molecular Biology, National Bioinformatics Infrastructure Sweden, Science for Life Laboratory, Uppsala University, Uppsala, Sweden; 4 National Bioinformatics Infrastructure Sweden, Science for Life Laboratory, Department of Biochemistry and Biophysics, Stockholm University, Stockholm, Sweden; 5 Department of Clinical Sciences Lund, Lund University, Lund, Sweden; 6 Department of Laboratory Medicine, Lund University, Lund, Sweden; 7 BioMS – Swedish National Infrastructure for Biological Mass Spectrometry, Lund University, Lund, Sweden; 8 National Public Health Laboratory, Bissau, Guinea-Bissau; 9 Nuffield Department of Medicine, University of Oxford, Oxford, UK

**Keywords:** Disease, Human specimen, Proteomics, Virology

## Abstract

Despite low or undetectable plasma viral load, people living with HIV-2 (PLWH2) typically progress toward AIDS. The driving forces behind HIV-2 disease progression and the role of viremia are still not known, but low-level replication in tissues is believed to play a role. To investigate the impact of viremic and aviremic HIV-2 infection on target and bystander cell pathology, we used data-independent acquisition mass spectrometry to determine plasma signatures of tissue and cell type engagement. Proteins derived from target and bystander cells in multiple tissues, such as the gastrointestinal tract and brain, were detected at elevated levels in plasma of PLWH2, compared with HIV negative controls. Moreover, viremic HIV-2 infection appeared to induce enhanced release of proteins from a broader range of tissues compared to aviremic HIV-2 infection. This study expands the knowledge on the link between plasma proteome remodeling and the pathological cell engagement in tissues during HIV-2 infection.

## Highlights

• Plasma proteome profiling of HIV negative, HIV-1, and HIV-2 infected individuals
• Secreted and leakage plasma protein signatures differ in HIV-1 and HIV-2 infection
• HIV-1 and HIV-2 viremia alter plasma protein profiles of target and bystander cells
• Protein signatures link bystander cell engagement and HIV-2 mediated inflammation

## Graphical abstract

**Figure:**
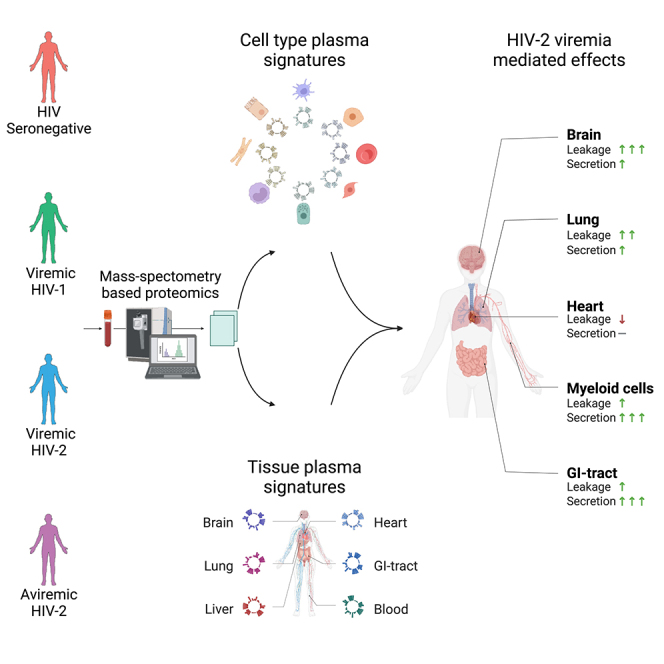

## Introduction

Human immunodeficiency virus type 1 (HIV-1) and type 2 (HIV-2) are the two causative agents of AIDS. Although they share several key characteristics, such as transmission route and clinical symptoms, HIV-2 has been shown to be less transmissible and pathogenic than HIV-1.^1^^,^^2^ Time to AIDS and HIV-related death are approximately twice as long in people living with HIV-2 (PLWH2), compared to HIV-1 (PLWH1).^3^ However, the underlying mechanism behind this difference is not known, although PLWH2 have been shown to mount a broader and more potent immune response compared to PLWH1.^4^^,^^5^^,^^6^^,^^7^^,^^8^^,^^9^^,^^10^^,^^11^ In line with this, the plasma viral load (pVL) of PLWH2 is generally lower compared with PLWH1 – both at setpoint, and when matched for CD4^+^ T-cell levels.^12^^,^^13^ Despite reduced viremia, most PLWH2 will develop AIDS if not treated with antiretroviral therapy (ART).^3^

Persistent viral replication, and its subsequent impact on e.g., mucosal integrity, is believed to be a major driver of chronic immune activation and exhaustion in PLWH1 (reviewed in^14^^,^^15^). This chronic immune activation and inflammation may also impact non-HIV susceptible cells, hereafter referred to as bystander cells.^16^^,^^17^^,^^18^^,^^19^ For example, in PLWH1, elevated plasma levels of proinflammatory cytokines can contribute to endothelial dysfunction, fibrotic remodeling, and hypercoagulation.^20^^,^^21^ Although the impact of the dysregulation of bystander cells have been extensively studied in PLWH1, in particular within the field of cardiovascular diseases,^22^ less is known about bystander cells in HIV-2 infection. Moreover, we and others have shown that both viremic and aviremic PLWH2 display signs of immune pathology, such as expansion of activated and exhausted CD4^+^ and CD8^+^ T-cells, B-cells, natural killer cells (NK-cells), NK T-cells (NKT), monocytes, and dendritic cells (DCs).^23^^,^^24^^,^^25^^,^^26^^,^^27^^,^^28^^,^^29^ Interestingly, HIV-2 RNA and Gag protein have been found in peripheral blood mononuclear cells and lymphoid tissue of aviremic PLWH2, indicating ongoing viral replication despite having undetectable plasma viral load.^30^^,^^31^

In this study, we used data-independent acquisition mass spectrometry (DIA-MS) to perform an in-depth characterization of the plasma proteome in HIV-1 and HIV-2 infections, with a particular focus on PLWH2. The specific objectives were to elucidate associations between plasma proteins, signatures of cell and tissue engagement, and pVL. The analysis showed that PLWH2, similar to PLWH1, had elevated plasma protein levels released from both target and bystander cells of different tissues. Furthermore, the results showed that plasma protein signatures of cell type perturbations in PLWH2 were associated with elevated inflammation, frequency of exhausted CD8^+^ T-cells, and pVL levels.

## Results

### Study participants

To investigate the impact of HIV-1 and HIV-2 infection on the blood plasma proteome, including both blood cell derived proteins and proteins originating from tissue resident cells, plasma samples were collected from 11 PLWH1, 23 PLWH2, and 27 HIV seronegative individuals. All PLWH1 were viremic, either naive to treatment (n = 4) or not successfully ART treated (n = 7), and the PLWH2 were either naive to treatment (n = 22) or not successfully ART treated (n = 1), and could be further divided into viremic (n = 9) or treatment-naïve aviremic (n = 14) based on the pVL quantification level of 75 HIV RNA copies/ml.^32^ Viremic PLWH1, viremic PLWH2, and aviremic PLWH2 had statistically lower median CD4^+^ T-cell percentage (CD4%) of all lymphocytes (10.7 [interquartile range (IQR) 6.4–12.6]; p < 0.001, 14.2 [IQR 11.2–14.4]; p < 0.001, and 33.4 [IQR 26.8–41.7]; p = 0.38, respectively) and absolute CD4^+^ T-cell count per μL (239 [IQR 139–293]; p < 0.001, 232 [IQR 146–421]; p < 0.001, and 636 [IQR 456–966]; p = 0.018, respectively) compared to the HIV seronegative individuals (39.3 [IQR 36.7–43.5] and 918 [IQR 862–1177], respectively). Additional characteristics of the study participants are described in Table 1.

**Table 1:** Characteristics of study participants

Characteristic	Viremic	Viremic	Aviremic	HIV
	PLWH1^a^	PLWH2^b^	PLWH2^c^	Seronegative
Numbers (female/male)	11 (4/7)	9 (0/9)	14 (5/9)	27 (10/17)
Age in years^d^	51 (46–53)	57 (53–59)	56 (48–60)	55 (48–60)
CD4^+^ T-cell% of lymphocytes^d^	11 (6–13)	14 (11–14)	33 (27–42)	39 (37–44)
CD4^+^ T-cells (cells/μL)^d^	239 (139–293)	232 (146–421)	636 (456–966)	918 (862–1177)
Viral load (copies/mm^3^)^d^^,^^e^	37322 (23079–54663)	4184 (1227–18216)	<75	NA

a Treatment naive or not successfully ART treated viremic (plasma VL > 1000 RNA copies/ml) HIV-1 infected individuals.

b Treatment naive (VL > 75 RNA copies/ml) or not successfully ART treated (plasma VL > 1000 RNA copies/ml) HIV-2 infected viremic individuals.

c Treatment naive aviremic HIV-2 infected individuals (plasma VL < 75 RNA copies/ml).

d Median (interquartile range).

e Plasma viral load quantification limit was 75 RNA copies/ml plasma for both HIV-1 and HIV-2; NA = not applicable.

### Both HIV-1 and HIV-2 infections result in altered plasma protein composition

During initial data quality control analysis using the NormalyzerDE package, the VSN normalization algorithm produced the lowest mean of ‘intragroup coefficients of variation of all replicate groups’, lowest mean of ‘intragroup Median Absolute Deviation across all replicate groups’ and mean of ‘intragroup Pooled Estimate of Variance across all replicate groups’ (Figure S1A). The quality control also indicated that neither sex, age, sample preparation order, order of nLC-MS/MS analysis (running order), nor suboptimal ART treatment were associated with any clear distortion or skewing of the raw data intensity (Figure S1B). The raw data intensity was therefore not corrected for the above factors prior to further analysis. In total, 1 331 unique plasma proteins were detected. Of these, 81 proteins were targeted by the depletion columns and were therefore excluded from further analysis (Table S1). 379 proteins (30%) had more than 20% protein missingness across samples, and these proteins were excluded from further analyses (Table S2). At group-specific comparisons of the remaining 871 proteins (Table S3), the plasma levels of 102 proteins were altered in PLWH1 or PLWH2 compared to HIV seronegative individuals. Five proteins were significantly higher, and 20 proteins significantly lower, among PLWH1 than HIV seronegative individuals (Figure S1C). In addition, the plasma levels of 39 proteins were significantly higher, and 52 proteins were significantly lower, among PLWH2 compared to HIV seronegative individuals (Table S1; Figure S1C). Both HIV-1 and HIV-2 infections were associated with elevated ADA2, B2M, CD163, FLT4, GOLM1, GRN, HLA-B, ICAM3, IGFBP2, LILRA3, MARCO, and VCAM1, and decreased H4C1 and H2AC4 plasma levels (Figure S1D). Furthermore, 11 proteins were only dysregulated among PLWH1 and 77 proteins only among PLWH2 (Table S2). According to the Interferome database,^33^ 21/25 proteins altered in plasma of PLWH1 have previously been reported to be regulated by type I or II interferon (IFN) signaling, and 75/91 proteins altered in plasma of PLWH2, and 13/14 proteins dysregulated in both PLWH1 and PLWH2 (Table S3; Figure S2). When comparing the plasma proteome of PLWH1 and PLWH2, no proteins remained significantly altered after correction for multiple testing (Table S4). To investigate the impact of HIV viremia on the plasma proteome, we compared viremic PLWH (combining PLWH1 and PLWH2) to aviremic PLWH2. Interestingly, only the plasma levels of ADA2, B2M, EXT2, and LCP1 were significantly higher among the viremic PLWH (adjusted p < 0.05), and no proteins were significantly lower among viremic PLWH compared to aviremic PLWH2 (Table S5). In contrast, 374 proteins were significantly increased or decreased among viremic PLWH compared to HIV seronegative individuals (Table S6), while no proteins differed significantly between aviremic PLWH2 and HIV seronegative individuals (data not shown).

**Figure 1:**
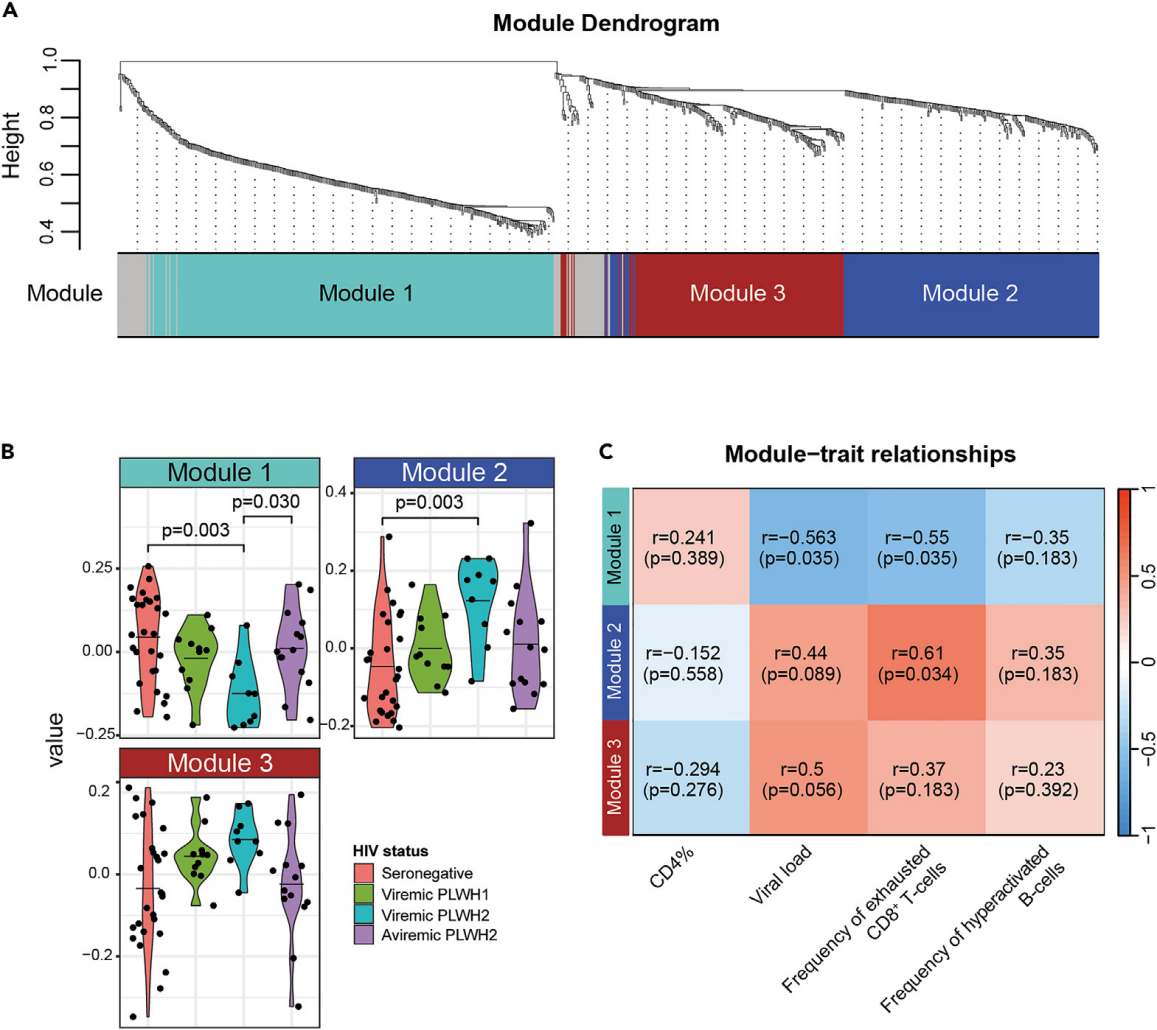
HIV-2 infection induces remodeling of the blood plasma proteome (A) Dendrogram illustrating clustering of analyzed proteins into different modules. (B) Comparisons of protein eigenvalues for each module between the different study groups, viremic PLWH1, viremic PLWH2, aviremic PLWH2 and HIV seronegative individuals. (C) Module – trait relationships, where Spearman Rank r- and p values are given for correlations between module protein eigenvalues and CD4^+^ T-cell percentage of all lymphocytes (CD4%), viral load, frequency of exhausted CD8^+^ T-cells, and frequency of hyperactivated B-cells, among PLWH2. Statistically significant group-wise differences were determined by ANOVA test, followed by Benjamini-Hochberg correction for multiple testing (p < 0.05). Statistically significant correlations were determined using the Spearman Rank correlation test, followed by BH correction for multiple testing (p < 0.05). Mean values are depicted in violin plots.

To gain a deeper insight into processes associated with the remodeling of the plasma proteome in HIV-1 and HIV-2 infections, we performed a weighted protein co-expression network analysis (WPCNA) to determine clusters, or modules, of highly correlated proteins. Three modules were identified, containing 339, 232, and 191 proteins, respectively (Figures 1A and S3A; Table S7). Proteins that did not match to either of the three modules were excluded from downstream analyses. To determine how the modules were associated with HIV-1 and HIV-2 infections, the expression profile of each module was summarized by protein eigenvalues, estimated by principal component analysis. Group-wise comparisons showed that module 1 was significantly reduced (p = 0.003), and module 2 increased (p = 0.003) in viremic PLWH2 compared to HIV seronegative individuals (Figure 1B). Among the top ten connected hub proteins in module 1, all were found to be present at lower plasma levels among viremic PLWH2 compared to HIV seronegative individuals (Figure S4A). Furthermore, nine of the top ten hub proteins in module 2 were found to be elevated among viremic PLWH2 compared to HIV seronegative individuals (Figure S4B). The plasma levels of three of these proteins, LAMB1, LRP1, and ALCAM, were only elevated among PLWH2 in the group-specific comparisons of all 871 proteins as well. In contrast, no statistically significant difference between the study groups was observed for the top ten hub proteins of module 3 (results not shown). To gain insight into the biological processes associated with the proteins in the modules, a gene ontology (GO) analysis was performed for each module. These indicated that module 1 contained proteins involved in actin filament organization, and module 2 proteins involved in cell adhesion (Figure S3B). Further analysis indicated that module 1 was inversely associated with the pVL (r = −0.563, p = 0.035), and that both modules 1 and 2, were associated with the frequency of exhausted CD8^+^ T-cells (r = −0.550, p = 0.035; r = 0.608, p = 0.034, respectively), among PLWH2 (Figure 1E). Taken together, these findings suggest that HIV-2 infection induce alterations in the composition of the plasma proteome that could be linked to actin polymerization and cell-adhesion.

### Both HIV-1 and HIV-2 infections induce tissue protein leakage

Since we identified increased plasma levels of proteins involved in cell adhesion in both PLWH1 and PLWH2, we hypothesized that this was due to increased egress of immune cells into peripheral tissues. We therefore utilized a previously described tissue-specific transcriptional signature dataset,^34^ to assess tissue damage and perturbations (hereafter referred to tissue engagement) that may be associated with HIV-1 and HIV-2 infection. In total, 337 of the 871 detected proteins overlapped with the tissue-specific transcriptional signature dataset. To specifically assess tissue engagement related to tissue resident cells, we removed proteins previously described to be enriched among whole blood cells.^34^ The predicted location of proteins, including proteins predicted to be actively secreted into blood plasma (hereafter referred to as secreted proteins) was extracted from the Human Protein Atlas (HPA).^35^^,^^36^^,^^37^ The remaining proteins were annotated as leakage proteins and included proteins that had leaked into the blood from damaged tissues or cells. Accordingly, we divided the dataset into secreted (n = 139) and leakage (n = 198) proteins to gain a deeper insight into the impact of HIV-1 and HIV-2 infections (Table S8). The median of the normalized protein intensity of all proteins in each tissue-specific transcript list was used as the tissue score for that specific tissue. In total, 36 and 28 tissue scores were created for leakage and secreted proteins, respectively (Figure S5A). The identified tissues included known HIV-1 replication sites, such as the sigmoid colon, lung, and spleen. The total number of proteins in each tissue ranged from 1 to 34 for leakage proteins and from 1 to 90 for secreted proteins (Figure S5A).

**Figure 2:**
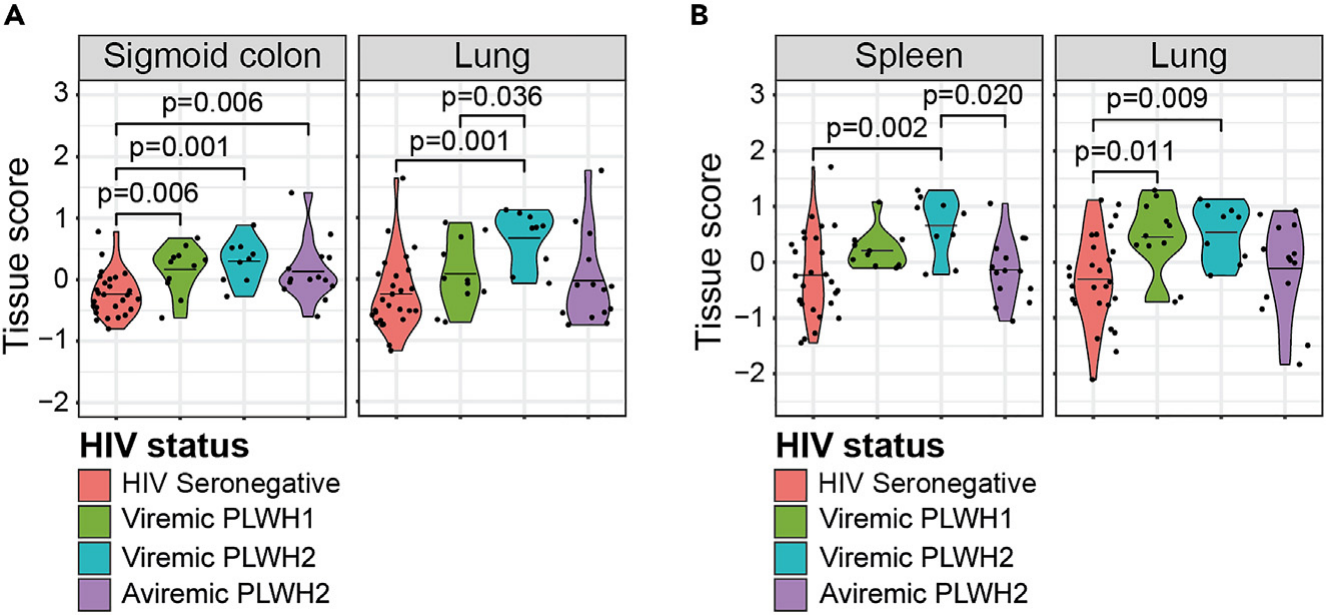
Both HIV-1 and HIV-2 infections induces increased release of proteins enhanced from known HIV-1 replication sites Comparisons between study groups, i.e., viremic PLWH1, viremic PLWH2, aviremic PLWH2, and HIV seronegative individuals, with respect to (A) leakage protein-derived tissue signatures, and (B) secreted protein-derived tissue signatures. Statistically significant group-wise differences were determined by ANOVA test, followed by Benjamini-Hochberg correction for multiple testing (p < 0.05). Mean values are depicted in violin plots.

Based on the leakage proteins, viremic PLWH1, viremic PLWH2, and aviremic PLWH2 had significantly higher sigmoid colon tissue scores compared to HIV seronegative individuals (p = 0.006, p = 0.001, and p = 0.006, respectively, Figure 2A). Specifically, the sigmoid colon signature score was positively associated with the established marker of microbial translocation, the lipopolysaccharide (LPS) co-receptor sCD14,^38^ in PLWH2 (r = 0.544, p = 0.016, Figure S5B). In addition, viremic PLWH2 had elevated lung tissue scores compared to both HIV seronegative and viremic PLWH1 (p = 0.001 and p = 0.036, respectively, Figure 2A). Alterations among other tissues, including heart and brain, were also found comparing the different study groups (Figure S5C). When analyzing the tissue scores created using the secreted proteins, both viremic PLWH1 and PLWH2 had elevated lung tissue scores compared to HIV seronegative individuals (p = 0.011 and p = 0.009, respectively, Figure 2B). The viremic PLWH2 also had higher spleen tissue score compared to HIV seronegative individuals (p = 0.002) and aviremic PLWH2 (p = 0.020), respectively (Figure 2B). No significant differences were observed between PLWH1 and PLWH2 for these two tissues. However, significant differences among the PLWH1, PLWH2, and HIV seronegative individuals, were found in additional tissues, such as the cardiovascular tissue (Figure S5D). Taken together, these results suggest that both HIV-1 and HIV-2 infections induce engagement of three known replication sites.

### HIV-2 infection induces perturbations of target and bystander cells in multiple tissues

**Figure 3:**
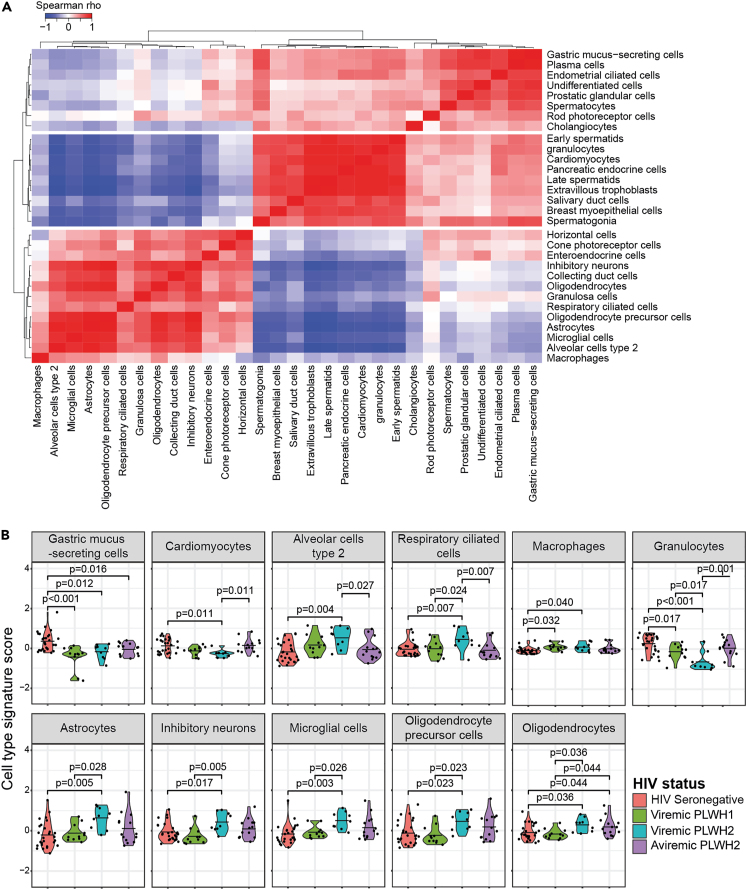
Viremic HIV-2 infection induced increased leakage of multiple cell types from known virus replication sites (A) Relationships and (B) groupwise comparisons of leakage protein-derived cell types. Statistically significant correlations were determined using the Spearman Rank correlation test, followed by Benjamini-Hochberg (BH) correction for multiple testing (p < 0.05) and group-wise differences were determined by ANOVA test, followed by BH correction for multiple testing (p < 0.05). Mean values are depicted in violin plots.

Although the tissue signature analysis suggested engagement of several tissues by HIV-1 and HIV-2 infections, it did not provide information about which cell types that were impacted by the infections. To assess this, we inferred the cell origin of the detected proteins based on the distribution of gene expression across cell types as defined in the HPA.^36^^,^^39^ In total, 674 of the 871 detected proteins were defined as cell type enhanced. We divided the dataset into secreted (n = 222) and leakage (n = 452) proteins. Signatures from 74 to 79 cell types were created using the secreted and leakage proteins, respectively (Table S9). The number of proteins in each cell type signature ranged from 1 to 117 for the secreted proteins, and 2–57 for the leakage proteins (Figure S6). The analysis suggested that the leakage plasma signature for 30 cell types were altered in HIV-1 and/or HIV-2 infections (Figures S7A and S5B). To identify associations between the cell type scores that differed significantly between the study groups, agglomerative hierarchical clustering based on the correlation of the cell type signature scores across all study participants was used (Figure 3A). The clustering indicated three groups of associated cell type signatures, and the signatures in group 1 and group 2 were inversely correlated with each other. Group 1 contained two known HIV target cells, macrophages and microglia cells, as well as tissue resident bystander cells from two tissues where these cells can be found, the lung and brain. Group 2 and 3 contained a mixture of cells from a broad range of tissues. When examining the individual cell type signatures scores, we found that cell types from several organs had elevated plasma signatures among viremic PLWH2, compared to HIV seronegative individuals (Figure 3B). These included astrocytes (p = 0.005), inhibitory neurons (p = 0.017), microglia cells (p = 0.003), oligodendrocyte precursor cells (p = 0.023), and oligodendrocytes (p = 0.036) from the brain; alveolar type 2 cells (p = 0.004), and respiratory ciliated cells (p = 0.007) from the lung; and macrophages (p = 0.040) from the innate immune system. In contrast to the elevated signature scores, the cardiomyocyte (p = 0.011), gastric mucus-secreting cell (p = 0.012), and granulocyte (p < 0.001) signatures were significantly lower among viremic PLWH2 compared to HIV seronegative individuals. The effect of aviremic HIV-2 infections was less pronounced, and of above-mentioned cell types, only the oligodendrocytes signature was elevated (p = 0.044), and the gastric mucus-secreting cell signature reduced (p = 0.016), compared to HIV seronegative individuals (Figure 3B). Among PLWH2, the macrophage signature was positively associated with the three inflammatory markers IP-10 (r = 0.712; p = 0.002), b2m (r = 0.682; p = 0.002), sCD14 (r = 0.597; p = 0.009), and the frequency of exhausted CD8^+^ T-cells (r = 0.541; p = 0.012) (Figure S7C). In contrast, the cardiomyocyte signature was inversely associated with the IP-10 (r = −0.513; p = 0.035), b2m (r = −0.475; p = 0.040), sCD14 (r = −0.468; p = 0.040), the frequency of exhausted CD8^+^ T-cells (r = −0.511; p = 0.035), and the pVL (r = −0.703; p = 0.001), respectively (Figure S7C).

**Figure 4:**
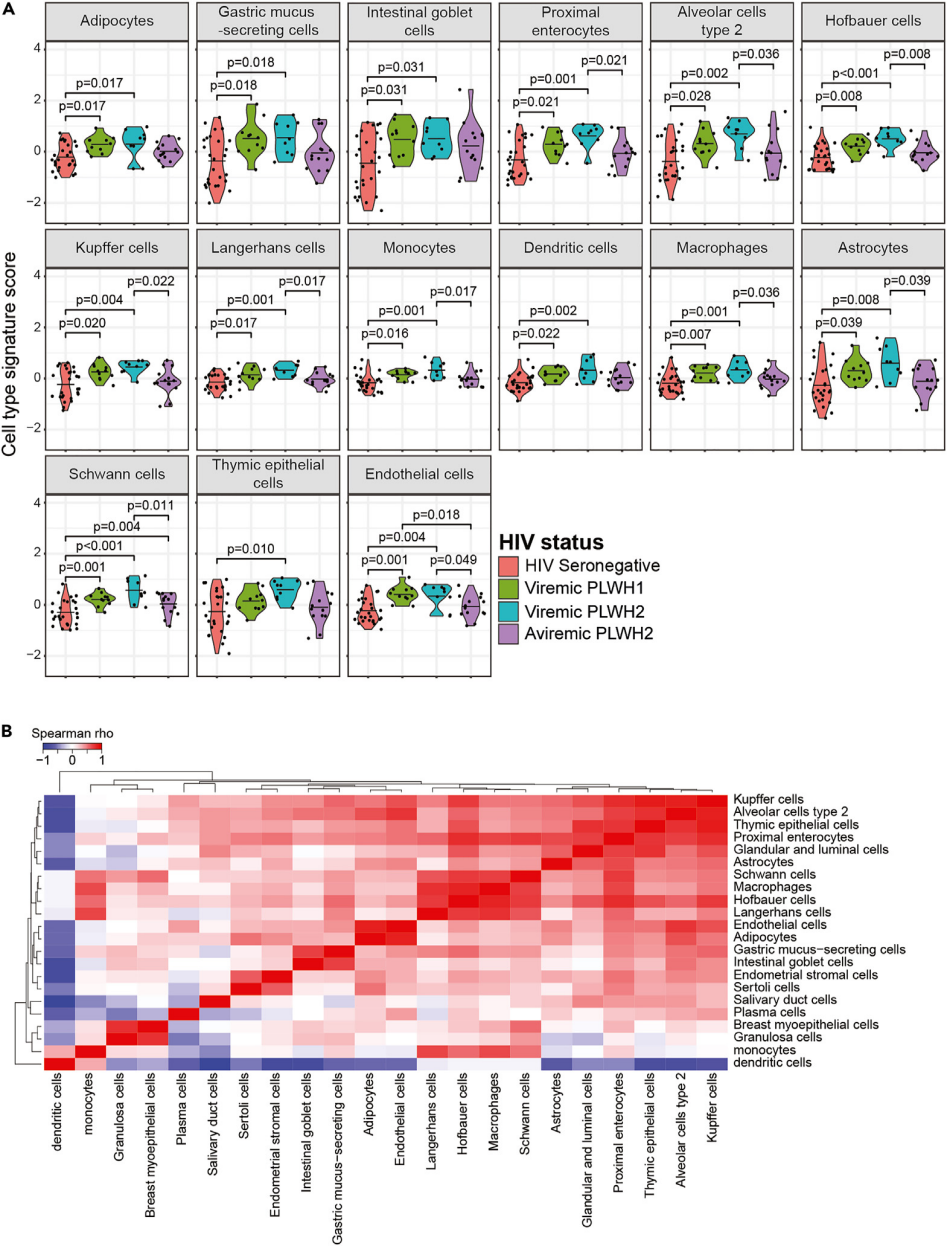
Viremic HIV-2 infection induced increased secretion of proteins of multiple target and bystander cells (A) Groupwise comparisons and (B) relationships between secreted protein-derived cell types. Statistically significant group-wise differences were determined by ANOVA test, followed by Benjamini-Hochberg (BH) correction for multiple testing (p < 0.05) and correlations were determined using the Spearman Rank correlation test, followed by BH correction for multiple testing (p < 0.05). Mean values are depicted in violin plots.

Next, we investigated associations between the study groups and the cell type signatures created with the secreted proteins. In total, 22 tissue signatures differed significantly between the groups (Figures 4A, S8A, and S8B). In contrast to the leakage protein-derived signatures, these 22 signatures were all increased in PLWH1 and/or PLWH2, compared to HIV seronegative individuals. Moreover, in contrast to the cell type signatures created using the leakage proteins, the secreted protein-based cell type signatures did not separate into clear groups based on hierarchical clustering of inter-cell type signature score correlations (Figure 4B). Only the dendritic cell signature was separated from the remaining 21 signatures. Similar to the results of the leakage-protein-derived cell type scores, the secreted protein-derived cell type signature scores of cells from several different tissues were higher among viremic PLWH2, compared to HIV seronegative individuals. These included adipocytes (p = 0.017) from adipose tissue; astrocytes (p = 0.008) and Schwann cells (p < 0.001) from the nervous system; alveolar type 2 cells (p = 0.002) from the lung; gastric mucus-secreting cells (p = 0.018), intestinal goblet cells (p = 0.031), and proximal enterocytes (p = 0.001) from the gastrointestinal tract; monocytes (p = 0.001), macrophages (p = 0.001), Kupffer cells (p = 0.004), dendritic cells (p = 0.002), and Langerhans cells (p = 0.001) from the innate immune system; and endothelial cells (p = 0.004) (Figure 4A). Interestingly, the thymic epithelial cells signature score was only significantly increased among viremic PLWH2 (p = 0.010), compared to HIV seronegative individuals. Of the above-described cell types, only the Schwann cells signature was elevated among aviremic PLWH2 compared to HIV seronegative individuals (p = 0.040). Instead, cell type signature in aviremic PLWH2 was significantly lower for ten cell types, compared to viremic PLWH2. In line with the observed positive correlation between the sigmoid colon tissue signature and sCD14 plasma concentration, the proximal enterocyte, endothelial cell, macrophage, monocyte, and Kupffer cell type signatures were all associated with IP-10, b2m, and sCD14, as well as the frequency of exhausted CD8^+^ T-cells and the pVL in PLWH2 (Figure S8C).

Taken together, these findings suggest that especially viremic HIV-2 infection induces perturbations of a wide range of different cell types. These included both target and bystander cells from tissues known to be impacted by HIV infection, such as the cardiovascular and nervous systems, as well as the gastrointestinal tract.

## Discussion

This in-depth characterization of the blood plasma proteome in PLWH suggests that viremic HIV-2 infection induces increased leakage of proteins from three replication sites, the gastrointestinal tract, the spleen, and the lung; as well as from several specific cell types found in these tissues. In addition, plasma signatures of several tissue resident cell types from other tissues were impacted in viremic HIV-2 infection. These findings therefore suggest that despite lower levels of viremia in PLWH2 compared to PLWH1, HIV-2 infection is associated with systemic pathological consequences.

To the best of our knowledge, no previous study has investigated the impact of untreated HIV-2 infection on the plasma proteome. As HIV-2 is primarily endemic in resource limited countries where routine pVL are not available to assess ART treatment success,^40^ there is a need for the identification of biomarkers that can be used during treatment monitoring. In addition, treatment monitoring is complicated in PLWH2 by the fact that many treatment naive PLWH2 are aviremic.^40^ We here used DIA-mass spectrometry to characterize the plasma proteome in PLWH1, PLWH2, and HIV seronegative individuals. The major strength of DIA-mass-spectrometry is the ability to detect hundreds of proteins in a non-targeted approach, making it suitable for both the discovery of novel biomarkers in HIV infection, and for increasing our understanding of HIV-induced pathology.^41^^,^^42^ We used network analysis, WPCNA, to gain a holistic understanding of HIV-induced alterations of the plasma proteome. This method was developed for analysis of transcriptomic data,^43^ but has recently also been used to analyze mass spectrometry based proteomics data to identify protein modules associated to clinical traits and biological processes.^44^^,^^45^^,^^46^^,^^47^^,^^48^ Gene ontology analyses are commonly performed to elucidate the common biological functions of the proteins found in each module. However, the blood plasma proteome contains both secreted and leakage proteins.^37^ The interpretation of gene ontology analyses of plasma proteins is therefore complicated by the fact that the biological function of an intracellular protein is not the same when it has leaked out into the extracellular space, or once a membrane bound receptor has been proteolytically cleaved and disassociated from the cell membrane.^37^^,^^49^ We therefore also investigated the possibility of using MS-based proteomics to assess perturbations of tissue and cell types. Indeed, plasma proteomics has recently been used to study multiorgan dysregulation and inter-organ communication in murine models, allowing for linkage between blood plasma and tissue pathology.^50^^,^^51^^,^^52^^,^^53^ To the best of our knowledge, data on large-scale proteomic profiling of human organs are not available, preventing similar studies from being performed based on human plasma. However, in two recent studies, the authors utilized the publicly available GTEx database to create tissue-enriched transcriptional signatures to study the impact of SARS-CoV-2 infection.^34^^,^^54^ In addition, two studies from the HPA program characterized tissue and single-cell RNA expression, with the support of immunohistochemistry, to create tissue and single cell expression maps.^36^^,^^39^^,^^55^ In the current study, we performed the first analysis of the contribution of HIV-1 and HIV-2 infections on tissue and cell type engagement by utilizing the GTEx and HPA program databases. A limitation of our approach was that the previous definition of tissue and cell type enhanced genes were defined by RNA sequencing, and not protein expression. Another limitation is the fact that cells and tissues included in the GTEx and HPA were only collected from healthy individuals. As HIV infections are known to induce large changes in the transcriptomic profile of many cell types, it is possible the transcriptomic profiles available at GTEx and HPA program databases do not accurately reflect the profiles in PLWH. However, several of the identified tissues and cell types have previously been reported to be involved in HIV pathogenesis,^22^^,^^56^^,^^57^ supporting the relevance of this approach. To gain further insight into the impact of HIV infections on tissues and cells, we divided the dataset into leakage and secreted proteins. The importance of this distinction was highlighted by the observation that signatures of the same cell type, such as gastric mucus-secreting cells, was significantly decreased for the leakage proteins, and significantly elevated for the secreted proteins. In addition, HIV infections were associated with increased secretion, but not leakage, of proteins from several additional myeloid cells and tissue resident cells found in the gastrointestinal tract. In contrast, the impact of HIV infection on neuronal cells were more obvious among the leakage protein-derived signatures compared to the secreted protein-derived signatures.

Analysis of tissue and cell type signatures suggested that both HIV-1 and HIV-2 infections were associated with increased release of proteins from the gastrointestinal tract. In agreement with that reported for PLWH1,^58^ we previously reported on the observation that PLWH2 have elevated LPS levels in plasma, i.e., microbial translocation, during advanced disease,^59^ suggestive of an impaired mucosal barrier in the gastrointestinal tract.^60^^,^^61^ Translocation of microbial products is believed to be a key driver of the chronic immune activation observed among PLWH.^15^^,^^60^ In line with this, we found that plasma level of sCD14, a well-established marker of microbial translocation and monocyte activation,^38^ was positively associated with signature scores of both tissues and cell types from the gastrointestinal tract. These included three myeloid cell types, monocytes, macrophages, and dendritic cells, as well as two gastrointestinal cell types, gastric mucus-secreting cells and proximal enterocytes. Enterocytes are epithelial cells that line the intestine and prevent translocation of microbial products from the lumen of the intestine into the intestinal wall.^15^ During HIV infections, enterocyte dysregulation can be induced both through interactions with virus proteins,^62^^,^^63^ and through bystander effects, such as infiltrating immune cells, such as monocytes/macrophages, and elevated inflammatory cytokine levels.^64^^,^^65^^,^^66^ More research is needed to identify drivers of intestinal tissue pathology in PLWH2, but a recent publication found the frequency of inflammatory monocytes to be positively associated with markers of bacterial translocation in PLWH2.^67^ In the current study, the proximal enterocyte signature was associated with the frequency of exhausted CD8^+^ T-cells, pVL, and plasma concentration of IP-10, sCD14, and b2m, suggesting that perturbation of enterocytes is associated with chronic inflammation in PLWH2. In addition to bystander effects from immune cells and proinflammatory cytokines, the positive association of the enterocyte signature score with pVL suggests that direct viral effects might play a role in enterocyte perturbation. In line with this, binding of HIV-1 to GPR15, an alternative co-receptor for HIV-1, has been suggested to induce enteropathy.^15^^,^^62^^,^^68^ The impact of HIV-2 binding to GPR15 on enterocytes has not been investigated, but as GPR15 represent a major co-receptor for HIV-2 viruses,^69^^,^^70^ it is possible that direct interactions between HIV-2 and enterocytes represent one of the underlying mechanisms behind HIV-induced enteropathy. Moreover, asymptomatic aviremic PLWH2 have been shown to have ongoing replication in the intestine and colon,^30^ suggesting that HIV-2 is produced in close proximity of enterocytes. Interestingly, plasma levels of the enterocyte enhanced protein ANPEP,^39^ one of the top ten connected hub proteins in the WPCNA module 2, was significantly higher among viremic PLWH2. As this protein can also be expressed in other tissues, more research is needed to evaluate if the increased release of ANPEP is solely a consequence of enterocyte pathology.

It is well-known that macrophages and monocytes can be infected by both HIV-1 and HIV-2,^71^ and may therefore constitute HIV reservoirs.^57^^,^^72^ Thus, the increased release of macrophage and monocyte enhanced proteins into the peripheral blood may constitute signs of HIV-2 replication in macrophages. However, the association of the signatures with both pVL and the inflammatory markers suggests that monocytes and macrophages are also activated due to the chronic inflammatory state of PLWH. In line with this, PLWH2 have been found to harbor increased frequencies of activated monocytes.^29^ Here, ADA2, CD163, GRN, and MARCO plasma levels were elevated among both PLWH1 and PLWH2 compared to HIV seronegative individuals, suggesting increased activation of myeloid cells during HIV-1 and HIV-2 infections.^73^^,^^74^ Aberrant activation of monocytes and macrophages are believed to be important drivers of tissue damage and tissue fibrosis in PLWH. We found that the secreted protein-derived tissue scores of proximal enterocytes and macrophages were positively associated. Previous studies have reported that pro-inflammatory monocytes are recruited into the intestinal mucosa, differentiate into pro-inflammatory tissue-resident macrophages, and drive persistent local inflammation and tissue injury in PLWH1.^66^^,^^75^^,^^76^

Cardiomyocyte and endothelial cell damage by HIV-1, resulting in cardiovascular disease, has been attributed to both direct viral effects and chronic inflammation.^21^^,^^56^^,^^77^ Thus, the elevated endothelial and reduced cardiomyocyte cell signature scores detected in viremic PLWH2, associated both with inflammation and pVL, may imply cardiomyocyte and endothelial damage.^36^^,^^39^ Microvascular endothelial cells have been shown to be potential targets for infection with certain HIV-1 and HIV-2 strains *in vitro*.^78^ However, chronic inflammation has been a more studied cause of endothelial damage in PLWH1.^21^^,^^22^^,^^56^^,^^79^ The risk for cardiovascular disease is, for example, still increased in PLWH1 receiving ART, and becomes even more pronounced with an aging infected population.^22^

The WPCNA suggested that viremic HIV-2 infection induced a reduction of proteins involved in actin filament organization. Although it is well established that HIV-1 and HIV-2 manipulates the actin networks in infected cells,^80^ the effect of HIV infection on extracellular actin levels are still not well described. HIV-1 infection can induce hyperactivation of the actin depolymerization factor cofilin in both infected cells and resting bystander CD4^+^ T-cells.^81^^,^^82^^,^^83^^,^^84^ During target cell infection, activation of cofilin increases actin dynamics, which in turn increases HIV-1 entry and transport to the nucleus.^80^ In addition, hyperactivation of cofilin has been associated with poor CD4^+^ T-cell recovery following initiation of ART, and impairment of T-cell migration.^81^^,^^82^^,^^85^ However, it is still not known whether increased actin depolymerization is linked to reduced extracellular release of actin and other proteins involved in actin filament organization, as observed in this study. Moreover, a reduction in intracellular levels of cytoskeletal components, including actin-associated factors, have been reported to occur concomitant with the production of high levels of HIV-1 particles.^86^ Still, the role of extracellular actin during HIV-1 and HIV-2 infections is not as well known. During tissue damage, filamentous actin (F-actin) is released and act as a damage associated molecular pattern.^87^ To prevent the multimerization of actin into F-actin, gelsolin and other proteins of the actin scavenger system dissociates actin complexes into monomeric actin.^88^ However, both actin and gelsolin were lower among viremic PLWH2 in this study. Although others have reported lower levels of gelsolin in several diseases,^89^^,^^90^ this is typically in response to increased levels of actin.^91^^,^^92^ More research is therefore needed to further elucidate the role of extracellular actin in HIV-2 infections. A second protein module, containing proteins involved in cell adhesion, distinguished viremic PLWH2 from HIV seronegative individuals. These results are in line with the findings from the group-wise comparison of all 871 proteins, where ICAM-3 and VCAM-1 plasma levels were increased among both PLWH1 and PLWH2, and ICAM-1 and ALCAM levels only among PLWH2. It is well-established that HIV-1 infection induce increased release of soluble adhesion molecules, such as ICAM-1, ICAM-3, and VCAM-1.^93^ This has been suggested to occur both as a consequence of migration of leukocytes across the endothelium, and as part of a negative regulator of additional transmigration by acting as decoy-receptors.^94^^,^^95^ It is therefore plausible that the increased plasma levels of soluble adhesion molecules observed among PLWH2 reflects increased egress of immune cells out of the blood system. As nine of the top ten connected hub proteins in protein module 2 were elevated among viremic PLWH2, these proteins could potentially be used to monitor treatment success of viremic PLWH2. However, as they were not clearly elevated among aviremic PLWH2, more research is needed to identify markers of treatment success of this patient subset.

In our study, all female PLWH2 were aviremic, suggesting that female PLWH2 control their HIV-2 infection better compared to male PLWH2. Similarly, female PLWH1 have been suggested to have a lower set-point pVL compared to male PLWH1.^96^ However, while findings from our cohort, and others, suggest that female PLWH2 progress slower to AIDS compared to male PLWH2,^3^^,^^97^ the same has not been observed among female PLWH1. The underlying mechanisms behind the difference in disease progression rate associated with sex, in a HIV type-dependent manner, is not known, but could potentially be explained by the fact that females mount stronger type I interferon responses compared to males at matched HIV-1 pVL.^96^ While the high pVL of PLWH1 has been suggested to lead to increased immunopathology among female PLWH1 compared to male PLWH1 at matched pVL levels,^96^ it is possible that the naturally low to undetectable pVL of PLWH2 provides an advantage for female PLWH2. This could be explained both by the fact that HIV-2 has been shown to be more interferon-sensitive than HIV-1, and that the increased induction of type I interferon responses by HIV-2 has been suggested to result in an improved priming of HIV-specific CD8^+^ T-cells.^98^^,^^99^^,^^100^ However, to the best of our knowledge, no study has performed a systematic comparison of the anti-HIV-1 and anti-HIV-2 immune responses in males and females. More research is therefore warranted to further elucidate associations between sex, pVL, inflammation, and anti-HIV responses in male and female PLWH2 to further our understanding of natural HIV control.

In summary, we generated DIA-MS data and utilized available databases to perform an in-depth analysis of the plasma proteome in HIV-1 and HIV-2 infection, and the impact that these respective infections have on target and bystander cells. We found that both viremic and aviremic PLWH2 display signs of tissue engagement, and perturbations of both target and bystander cells, although the impact was often more severe among viremic PLWH2. In addition, our analysis strategy using advanced bioinformatics, combining proteomics data with that of publicly available databases, represents a framework for studies of the systemic impact of infections and other pathologies.

### Limitations of the study

This study is not without limitations. For example, the observed tissue or cell damage in peripheral tissues could not be verified by biopsies, since tissue biopsies were not available from the study participants. In addition, the identified tissue- and cell type-enhanced genes were based on previously published datasets of RNA expression in healthy tissues or cells. It is therefore possible that these datasets may not fully reflect RNA expression in HIV infection. Finally, all viremic PLWH2 in this study were male, while the other HIV status subgroups contained both male and female study participants. The lack of female viremic PLWH2 is likely due to a combination of the fact that the study cohort is dominated by male participants, as well as to the slower disease progression rate of female compared to male PLWH2.^3^

## Consortia

Current members of the Sweden Guinea-Bissau Cohort Research (SWEGUB CORE) group include Babetida N’Buna, Antonio J. Biague, Ansu Biai, Cidia Camara, Joakim Esbjörnsson, Marianne Jansson, Emil Johansson, Sara Karlson, Jacob Lindman, Patrik Medstrand, Fredrik Månsson, Malin Neptin, Hans Norrgren, Angelica A. Palm, Gülsen Özkaya Sahin, Zacarias José da Silva, Zsofia I. Szojka.

## STAR★Methods

### Key resources table

**Table:** 

REAGENT or RESOURCE	SOURCE	IDENTIFIER
**Biological samples**		

Human plasma	This manuscript	N/A

**Chemicals, peptides, and recombinant proteins**		

High Select™ Top14 Abundant Protein Depletion Mini Spin Columns	Thermo Scientific	A36370
Urea	Merck	Catalog:U1250
Ammonium bicarbonate	Merck	Catalog: A6141
Dithiothreitol	Merck	Catalog: D0632
Iodoacetamide	Merck	Catalog: I6125
Trifluoroacetic acid	Merck	Catalog: 302031
80% Acetonitrile, 20% Water with 0.1% Formic Acid, Optima™ LC/MS Grade	Fisher Chemical	Catalog: LS122
Water with 0.1% Formic Acid, Optima™ LC/MS Grade	Fisher Chemical	Catalog: LS118
iRT Kit	Biognosys	Catalog: Ki-3002-2
Sequencing grade modified trypsin	Promega	Catalog: V511

**Other**		

Acclaim® PepMap 100 C18 nano trap column, 75 μm x 2 cm, 3μm, 100Å, nanoViper	Thermo Scientific	Catalog: 164535
Easy-spray PepMap RSLC C18 column, 75μm x 50cm, 2μm, 100Å, nanoViper	Thermo Scientific	Catalog: ES903
Megafuge 8R Clinical Centrifuge	Thermo Scientific	Catalog: 50144445
Mini Centrifuge	Thermo Scientific	Catalog: 75004061
VORTEX Genius 3	IKA	Catalog: 412-0098
MS vial	VWR	Catalog: 548-0028
MS cap (blue)	VWR	Catalog: 548-0815
MS insert	VWR	Catalog: 548-0310

**Deposited data**		

The mass spectrometry proteomics data have been deposited to the ProteomeXchange Consortium via the PRIDE partner repository.	This manuscript	Pride: PXD045155 (https://www.ebi.ac.uk/pride/archive/projects/PXD045155)
Code	This manuscript	https://github.com/NBISweden/SMS-6353-22-HIV1n2

**Software and algorithms**		

Spectronaut (v16.0.220606.53000)	Biognosis AG	https://biognosys.com/resources/spectronaut-a-groundbreaking-increase-in-identifications/
The R-project (v4.2.1)	R core team^101^	https://www.r-project.org/
*iq* R-package (v1.9.6)	Pham et al.^102^	https://cran.r-project.org/web/packages/iq/index.html
*vsn* R-package (v3.66.0)	Huber et al.^103^	http://www.bioconductor.org/packages/release/bioc/html/vsn.html RRID:SCR_001459
NormalyzerDE R-package (v1.16.0)	Willforss et al.^104^	https://bioconductor.org/packages/release/bioc/html/NormalyzerDE.html
FactoMineR R-package (v2.6)	Lê et al.^105^	https://cran.r-project.org/web/packages/FactoMineR/index.html RRID:SCR_014602
*limma* R-package (v3.54.0)	Ritchie et al.^106^	https://bioconductor.org/packages/release/bioc/html/limma.html RRID: SCR_010943
*WGCNA* R-package (v1.72-1)	Langfelder et al.^43^	https://cran.r-project.org/web/packages/WGCNA/index.html RRID:SCR_003302
*clusterProfiler* R-package (v 4.6.2)	Wu et al.^107^	http://bioconductor.org/packages/release/bioc/html/clusterProfiler.html RRID:SCR_016884
*ggplot2* R-package (v3.4.0)	Wickham et al.^108^	https://ggplot2.tidyverse.org/ RRID : SCR_014601
*EnhancedVolcano* package (1.16.0)	Blighe^109^	https://bioconductor.org/packages/EnhancedVolcano/RRID:SCR_018931
ComplexHeatmap Package, version 2.14.0	Gu et al.^110^	http://bioconductor.org/packages/release/bioc/html/ComplexHeatmap.html RRID: SCR_017270

### Resource availability

#### Lead contact

Further information and requests for resources and reagents should be directed to and will be fulfilled by the lead contact Marianne Jansson (marianne.jansson@med.lu.se).

#### Materials availability

This study did not generate new unique reagents.

#### Data and code availability

• The mass spectrometry proteomics data have been deposited to the ProteomeXchange Consortium^111^ via the PRIDE partner repository with the dataset identifier PXD045155 (https://www.ebi.ac.uk/pride/archive/projects/PXD045155).
• All original code has been deposited and can be assessed at: https://github.com/NBISweden/ SMS-6353-22-HIV1n2 upon request. DOIs are listed in the key resources table.
• Any additional information required to reanalyse the data reported in this paper is available from the lead contact upon request.

### Experimental model and study participant details

#### Study participants

The overall goal of this study was to characterise the plasma proteome for understanding of the systemic impact of HIV-1 and HIV-2 infections. For this purpose, treatment naïve or not successfully ART treated individuals, with pVL>1000 RNA copies/ml plasma,^112^ PLWH1 (n=11) and PLWH2 (n=23), as well as HIV seronegative (n=27) individuals (Table 1), were enrolled from an occupational cohort of police officers in Guinea-Bissau.^113^^,^^114^ The group of PLWH2 included both viraemic (n=9), and treatment naïve aviraemic (n=14) individuals, as subdivided according to the plasma viral load quantification level, 75 RNA copies/ml.

#### Ethics

The study was approved by the Ethical committee at Lund University, and the National Ethical Committee, Ministry of Public Health in Guinea-Bissau. Informed consent was obtained from all participants. All samples used in this study were collected according to the guidelines in the Declaration of Helsinki.

### Method details

#### Sample collection, CD4^+^ T-cell level and plasma viral load determinations

Blood was collected using EDTA vacutainer tubes (BD Biosciences), and plasma separated at the National Public Health Laboratory, Guinea-Bissau, shipped frozen to Sweden for long-term storage. In parallel with the plasma collection, absolute CD4^+^ T-cell counts and CD4^+^ T-cell percentages of lymphocytes were performed using the FACS Presto instrument (BD Biosciences).^26^ HIV-1 and HIV-2 pVLs were previously determined.^26^^,^^32^

#### Inflammatory marker quantification

The inflammatory markers Interferon gamma-induced protein 10 (IP-10, also known as CXCL10), b2-microglobulin (b2m), soluble CD14 (sCD14), previously linked with immune activation, inflammation, viremia, and disease progression in PLWH1 and PLWH2,^115^^,^^116^^,^^117^ were quantified in plasma from PLWH2 using the Magnetic Luminex assay (R&D Systems Inc.).^26^

#### Sample processing for mass-spectrophotometry

Prior to the sample preparation for the nano-scale liquid chromatographic tandem mass spectrometry (nLC-MS/MS) analysis, the 14 most abundant proteins (Albumin, alpha-1-acid glycoprotein, alpha-1-antitrypsin, alpha-2-macroglobulin, apolipoprotein A1, fibrinogen, haptoglobin, and transferrin, and the kappa and lambda light chains of immunoglobulin (Ig) G, IgA, IgM, IgD, and IgE) were depleted from four microlitre plasma using the High Select™ Top14 Abundant Protein Depletion Mini Spin Columns (ThermoFisher Scientific), according to the manufacturer’s instructions. The depleted samples were thereafter prepared using a filter-aided sample preparation (FASP) approach. Briefly, the depleted plasma was transferred to Amicon 10 kDa filters (Merck Millipore), concentrated, denatured in 100 mM ammonium bicarbonate with 8M urea (Merck), reduced in 35mM dithiothreitol (Merck) for one hour at 37°C, and alkylated with 55mM iodoacetamide (Merck) for 30 minutes at room temperature in the dark. The proteins were subsequently washed twice with 100 mM ammonium bicarbonate before elution in 100mM ammonium bicarbonate. The protein concentration was determined using Qubit 4 Fluorometer (ThermoFisher Scientific), and the Invitrogen Qubit Protein BR Assay Kit (ThermoFisher Scientific). One μg of sequencing Grade Modified Trypsin (Promega) was added to 30 μg protein, and the samples were digested at 37°C for 16 hours. The digestion was stopped using 10% trifluoroacetic acid, and the peptide concentration was thereafter determined using a Nanodrop 2000 spectrophotometer (ThermoFisher Scientific). Finally, the samples were stored at -20°C, prior to nLC-MS/MS analysis.

#### Mass spectrometry analysis and data extraction

Prior to the nLC-MS/MS analysis, the samples were spiked with indexed retention time peptides. One μg of peptide solution per sample were injected onto the nLC column. The nLC-MS/MS analysis was performed on a Dionex Ultimate 3000 Rapid Separation LC (RSLC) nano Ultra-performance LC system coupled to an Orbitrap Exploris 480 MS with FAIMS Pro interface (Thermo Scientific). Peptides were loaded onto an Acclaim PepMap 100 C18 (75 μm × 2 cm, 3 μm, 100 A, nanoViper) trap column and separated on an Easy-spray PepMap RSLC C18 column (75 μm × 50 cm, 2 μm, 100 A) (Thermo Scientific) using a flow rate of 300 nL/min, and a column temperature of 60°C. A 90 min gradient was applied for separation, using solvents A (0.1% formic acid) and B (0.1% formic acid in 80% acetonitrile), increasing solvent B from 5% to 25% in 75 min, then to 32% in the next 9 min, and to 45% in following 6 min. Finally, the gradient increased to 95% solvent B for 2 min, and then continuing at 95% for another 5 min.

For DIA, full MS resolution was set to 120000 (at 200 m/z), AGC target value was 300% with a maximum injection time (IT) of 45 ms. Full MS mass range was set to m/z 380-1100. MS2 scans were acquired with a resolution of 30000 (at 200 m/z), fragmentation with a normalized collision energy (NCE) of 32, AGC target value was set to 1000% with automatic IT. Twenty-six variable isolation windows of 35.0, 22.5, 35.0 and 50.0 Da were used with an overlap of 0.5 Da, and with 1, 20, 1, 4 loop counts, respectively. For FAIMS, two compensation voltages (CVs) of -45 and -60 V were used with a carrier gas flow of 3.5 L/min and the spray voltage was set to 2.1 kV.

For data dependent acquisition (DDA) experiments (library generation), full MS resolution was set to 120000 (at 200 m/z), AGC target value was 300% with a maximum IT of 45 ms. Full MS mass range was set to 350-1400 m/z. MS2 scans were acquired with a resolution of 30000 (at 200 m/z), fragmentation with a NCE of 30, AGC target value was set to 100% with automatic IT. The precursor isolation window was set to 1.6 m/z, intensity threshold was kept at 2e^4^, and 45 s dynamic exclusion was used. For FAIMS CVs of -45 and -60 V were used with cycle times of 1.7 and 1.3 s, respectively.

#### Construction of spectral library for the DIA-MS analysis

We created a combined spectral library from DIA measurements of the individual plasma samples, and from DDA measurements of a sample pooled from 19 depleted plasma samples and fractionated by off-line high-pH reversed-phase chromatography (Pierce High pH Reversed-Phase Peptide Fractionation Kit, Thermo Fisher Scientific). The spectral library was generated using the Pulsar search engine incorporated into Spectronaut 16.0.220606.53000 (Biognosys, Schlieren, Switzerland) according to the factory default settings. All generated raw data were searched against the UniProtKB reviewed human database, including isoforms (accessed on May 27, 2022; 42,363 entries) combined with the HIV-1 and HIV-2 reference proteomes (accessed on May 27, 2022; 189 entries).^118^ The parameters included trypsin as digestion enzyme, two missed cleavages allowed, carbamidomethyl cysteine as fix modification, and methionine oxidation and acetylation on protein N-terminal as variable modifications. The number of identifications was controlled by false discovery rate (FDR) of 1% at peptide, and protein level, respectively. The spectral library consisted of 23581 precursors, 16075 peptides, 2427 proteins, and 1510 protein groups.

#### DIA-MS targeted data extraction

DIA data files were searched against the spectral library using the Biognosys (BGS) factory default settings in Spectronaut 16.0.220606.5300 (Biognosys, Schlieren, Switzerland). The number of identifications were filtered at an FDR of 1% at both peptide and protein levels.

### Quantification and statistical analysis

#### Data processing and quality control

The protein abundance was determined from peptide precursor and fragment ion intensities using the *iq* R-package (v1.9.6) in R (v4.2.1).^101^^,^^102^ The quantification data for the remains of the 14 top abundant proteins were excluded from the data set, as they could not be reliably quantified after the depletion. In accordance with the consensus in the field, proteins with more than 20% missingness were excluded from the analysis.^119^ For the remaining proteins with missing values, a random intensity value between one and the lowest quantified value was imputed. The protein intensity data was thereafter normalised by the variance stabilizing normalization (VSN) algorithm using the *vsn* R-package (v3.66.0).^103^

#### Statistical analyses

All statistical analyses were performed using R (v4.2.1).^101^

##### Data quality control

The NormalyzerDE R-package (v1.16.0) was used to determine which normalisation algorithm best separate the PLWH1, PLWH2, and HIV seronegative study groups.^104^ The dimensionality of the protein abundance data reduced by performing a principal component analysis (PCA) using the *FactoMineR* R-package (v2.6).^105^ The contribution of sample age, donor age, donor sex, percentage CD4^+^ T-cells of all lymphocytes at sample date, and HIV status, to the first five components was evaluated by a Pearson correlation test using the *stats* package (v4.2.1).^101^

##### Differential protein intensity analyses

Normalized protein intensity values were compared between the three different HIV status groups by linear regression analyses with sample age as a covariate using the *limma* R-package (v3.54.0).^106^ P-values were corrected for multiple testing by the Benjamini-Hochberg’s (BH) method using the *limma* R-package. Adjusted p-values <0.05 were considered significant.

##### Identification of interferon regulated proteins

Protein regulated by type I, type II, or both type I and type II interferons were identified using the Interferome database V2.01 (http://www.interferome.org/interferome/home.jspx).^33^ The 102 proteins found to be significantly altered among PLWH1 and PLWH2 were submitted to the database to identify interferon regulated proteins, and the specific regulating IFN type.

##### Weighted protein co-expression network analysis

A weighted protein co-expression network analysis (WPCNA) was performed using the *WGCNA* R-package (v1.72-1).^43^ A signed network was constructed using the blockwiseModules function with following settings: soft threshold power of 8, deepSplit value of 2, minimum module size of 30, and merge cut height of 0.25. Hub proteins (proteins that has the maximum connections with other proteins in a module) were obtained using the exportNetworkToVisANT function in the *WGCNA* R-package.^43^ Enriched biological processes in the modules was determined using the enricher function in the *clusterProfiler* R-package (v 4.6.2).^107^^,^^120^

##### Tissue and cell type analyses

A previously described tissue-enriched transcriptional signature dataset was recently created using the Genotype-Tissue Expression (GTEx) database.^34^ Briefly, they converted GTEx-acquired read counts to trimmed mean of M values, normalized the values to z-scores for each gene across tissues, and considered tissues enriched for genes if the z-score was above three. The cell type origin was assigned to proteins based on previous annotation performed by the Human Protein Atlas (HPA).^37^ To determine tissue and cell type signature scores, we converted the intensity values of proteins expressed from tissue or cell type enriched genes into z-scores based on the mean protein value across all donors. The individual tissue scores for each donor were defined as the mean z-score value of the proteins in each tissue gene list.^54^ The dataset was thereafter divided based on previously annotated localization, i.e., into either actively secreted to blood or not actively secreted to blood.^35^^,^^36^^,^^37^

##### Data visualization

The *ggplot2* R-package (v3.4.0) was used to produce the plots displaying PCA coordinates, violin plots displaying disease progression values, and tissue scores.^108^ The volcano plots were produced using the *EnhancedVolcano* R-package (1.16.0).^109^ Heatmaps were produced using the *ComplexHeatmap* R-package (v2.14.0).^110^

## Supplemental information

Document S1. Figures S1–S8

Table S1. Summary of protein intensity between people living with HIV-1 or HIV-2, compared to HIV seronegative individuals, related to Figures 1 and S1

Table S2. Differentially expressed proteins among PLWH1, PLWH2, or both PLWH1 and PLWH2, related to Figures 1 and S1

Table S3. Description of differentially expressed proteins regulated by interferon signaling, related to Figures 1 and S2

Table S4. Comparison of plasma protein levels between PLWH1 and PLWH2, related to Figures 1 and S1

Table S5. Comparison of plasma protein levels between viremic PLWH (PLWH1 and PLWH2) to aviremic PLWH2, related to Figures 1 and S1

Table S6. Comparison of plasma protein levels between viremic PLWH (PLWH1 and PLWH2) to HIV seronegative individuals, related to Figures 1 and S1

Table S7. Description of proteins in each WPCNA module, related to Figure 1

Table S8. Description of proteins in each tissue signatures, related to Figure 2

Table S9. Description of proteins in each cell type signature, related to Figures 3 and 4
