# Supplementary material for: HIV-2 mediated effects on target and bystander cells induce plasma proteome remodeling
Source: iScience. 2024 Feb 28;27(4):109344. doi: 10.1016/j.isci.2024.109344 (PMC10945182; doi:10.1016/j.isci.2024.109344)
Supplement: Document S1. Figures S1–S8 [file mmc1.pdf]

iScience, Volume *27*

Supplemental information

HIV-2 mediated effects on target and bystander cells induce plasma proteome remodeling

Emil Johansson, Jamirah Nazziwa, Eva Freyhult, Mun-Gwan Hong, Jacob Lindman, Malin Neptin, Sara Karlson, Melinda Rezeli, Antonio J. Biague, Patrik Medstrand, Fredrik Månsson, Hans Norrgren, Joakim Esbjörnsson, Marianne Jansson, and for the SWEGUB CORE group

Supplemental Figure S1

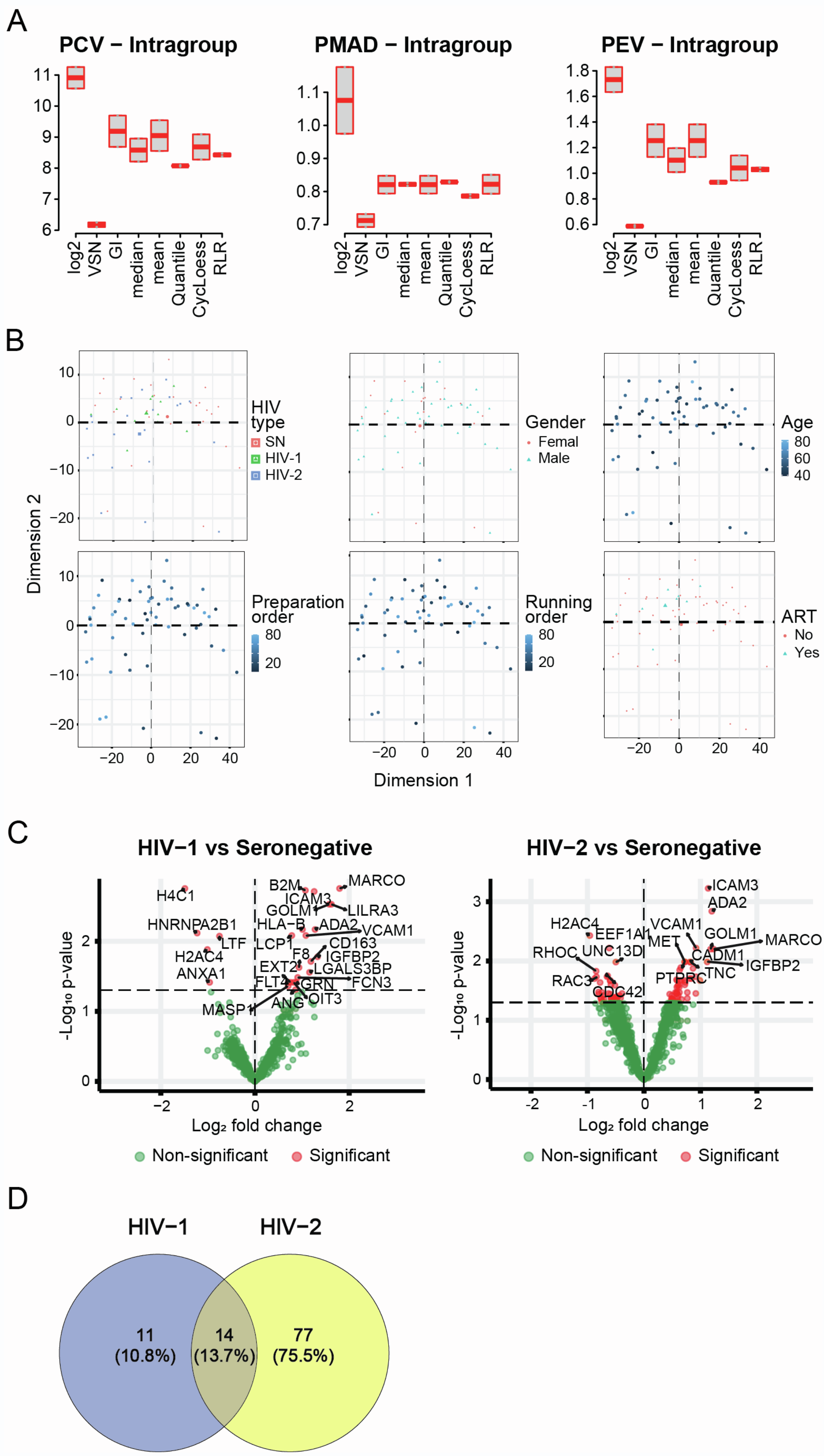

Supplemental Figure S2

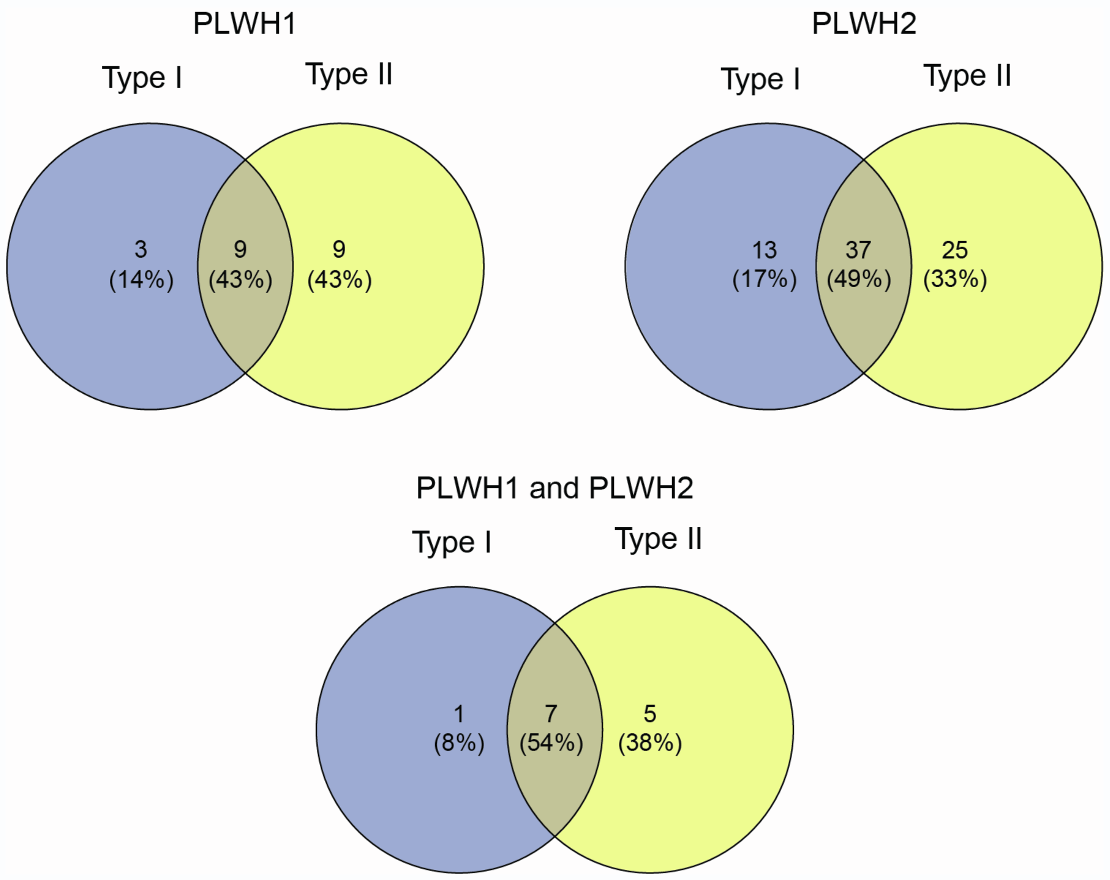

Supplemental Figure S3

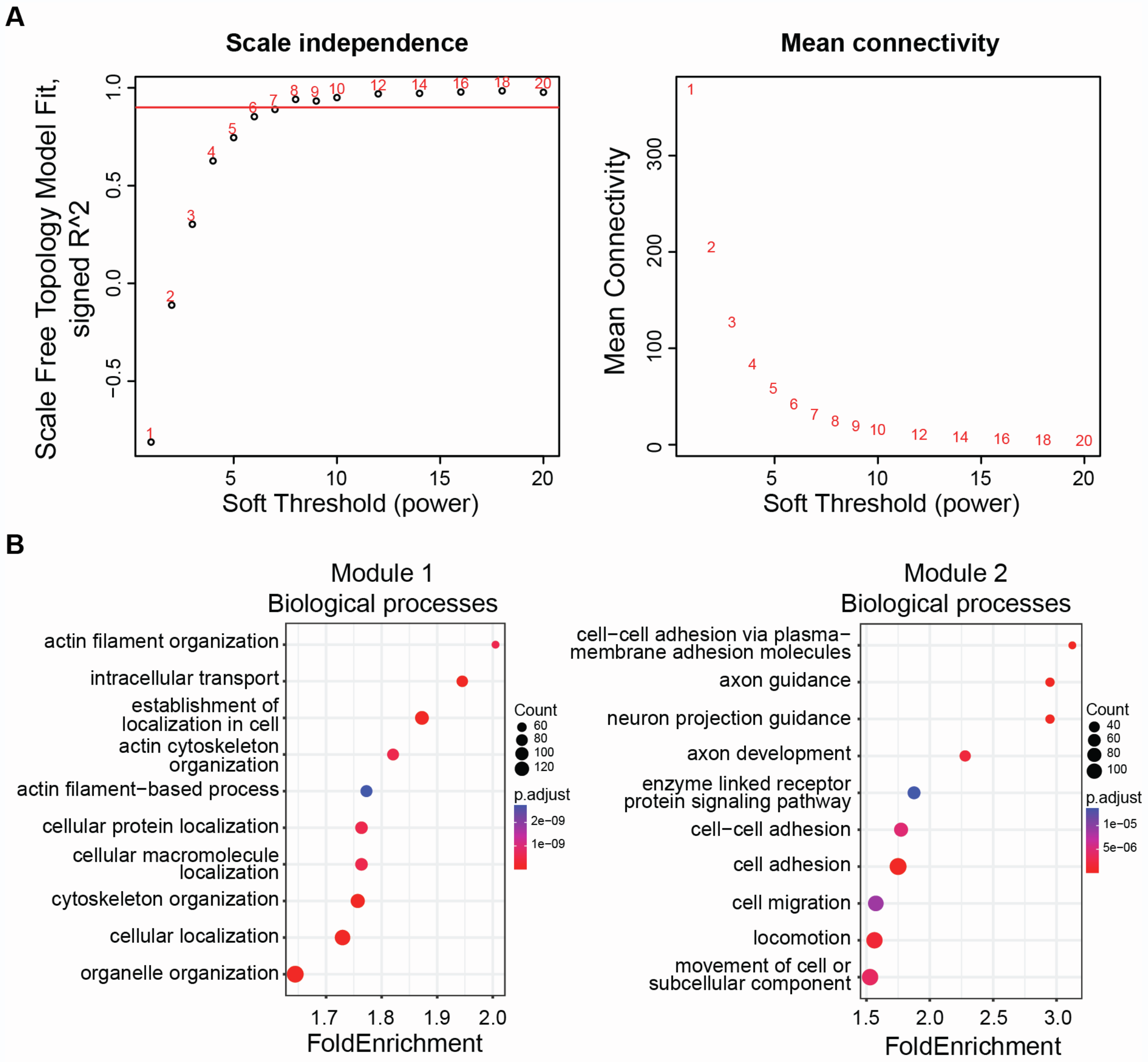

Supplemental Figure S4

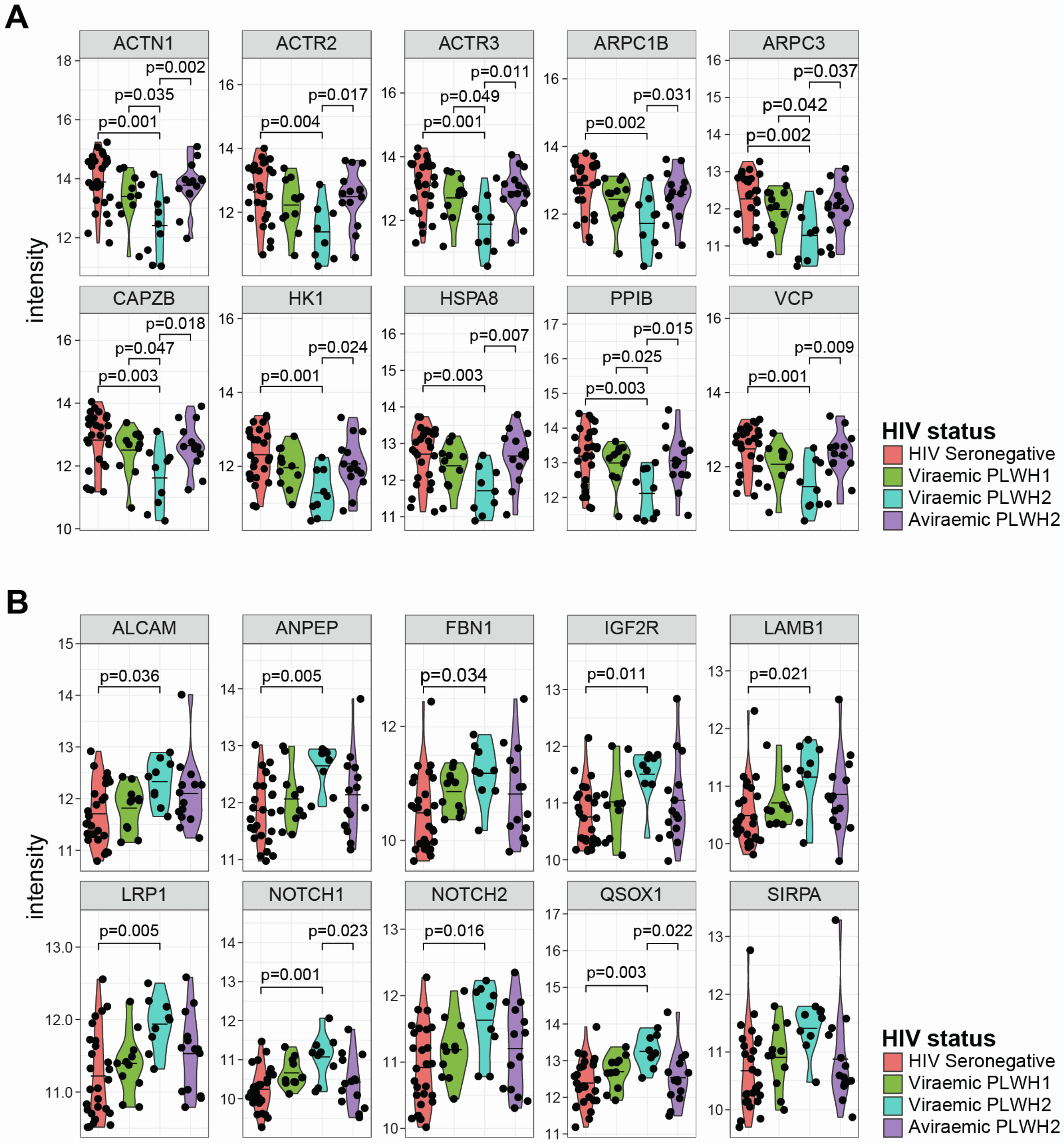

Supplemental Figure S5

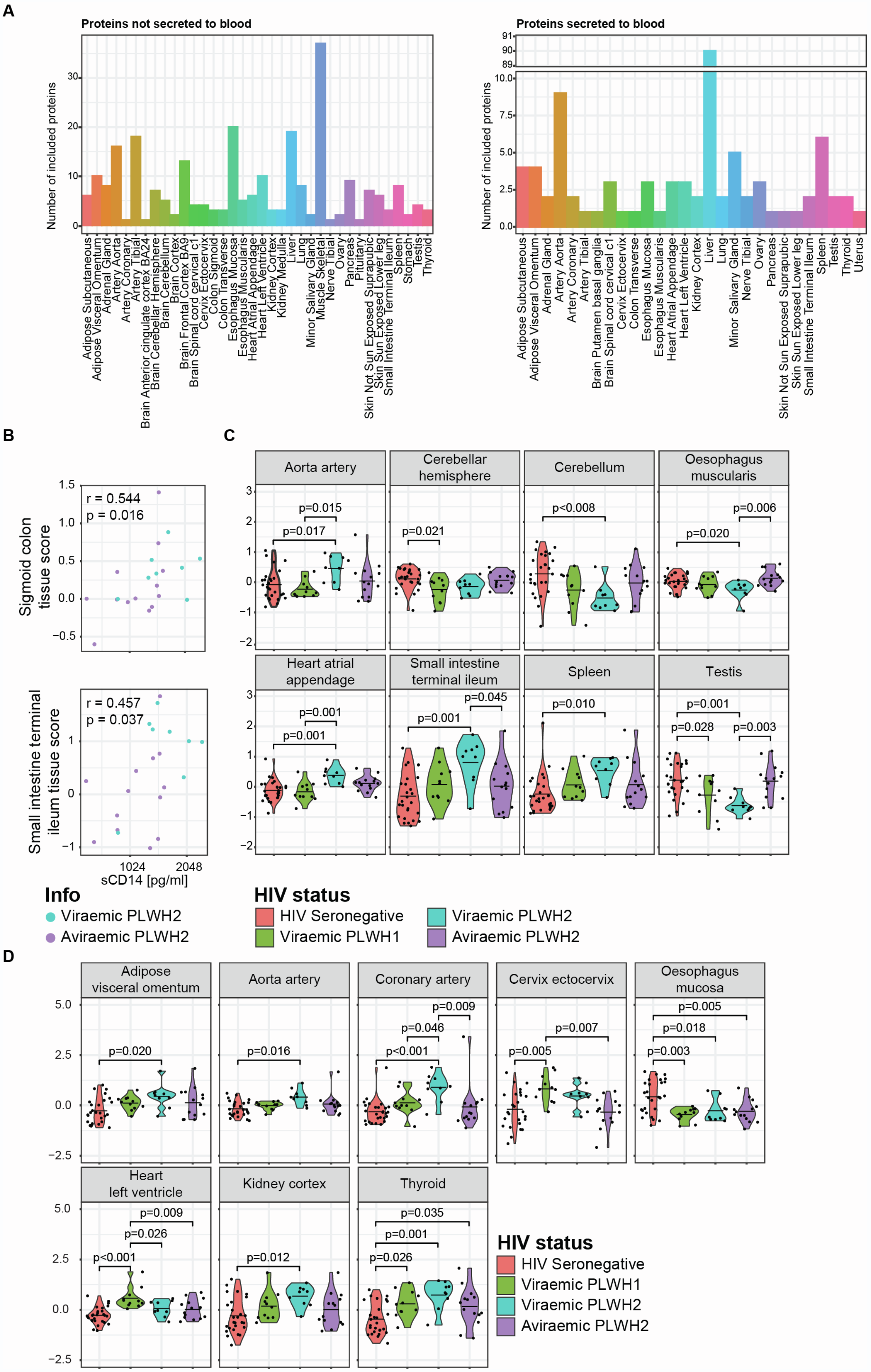

Supplemental Figure S6

Leakage protein-derived signatures

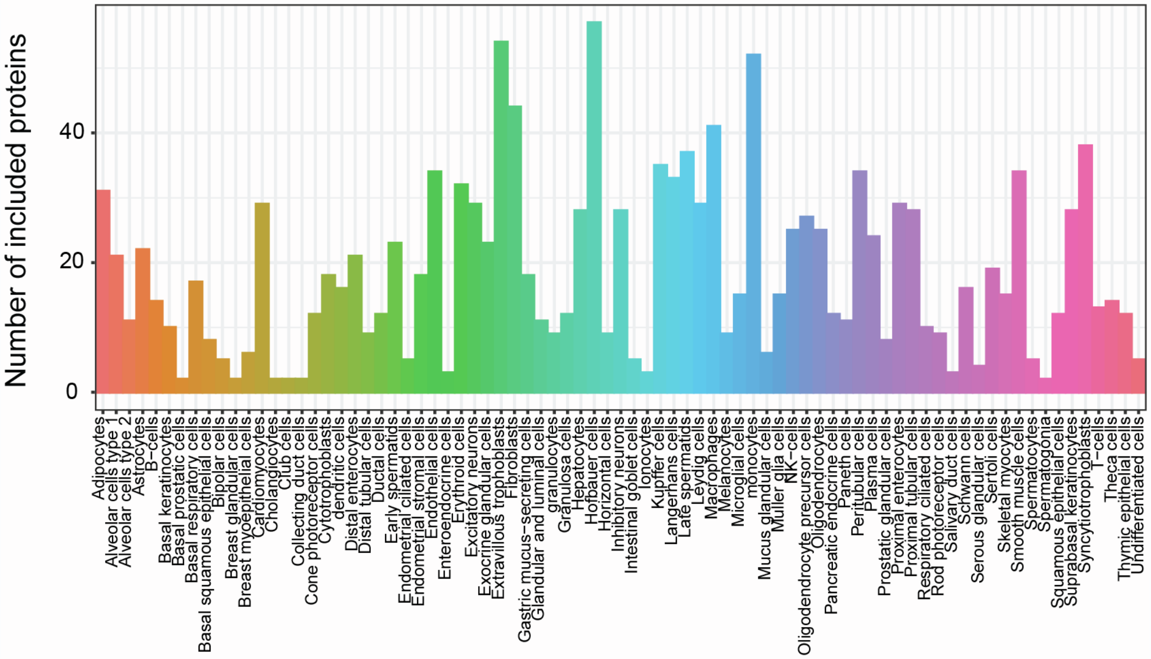

Secretion protein-derived signatures

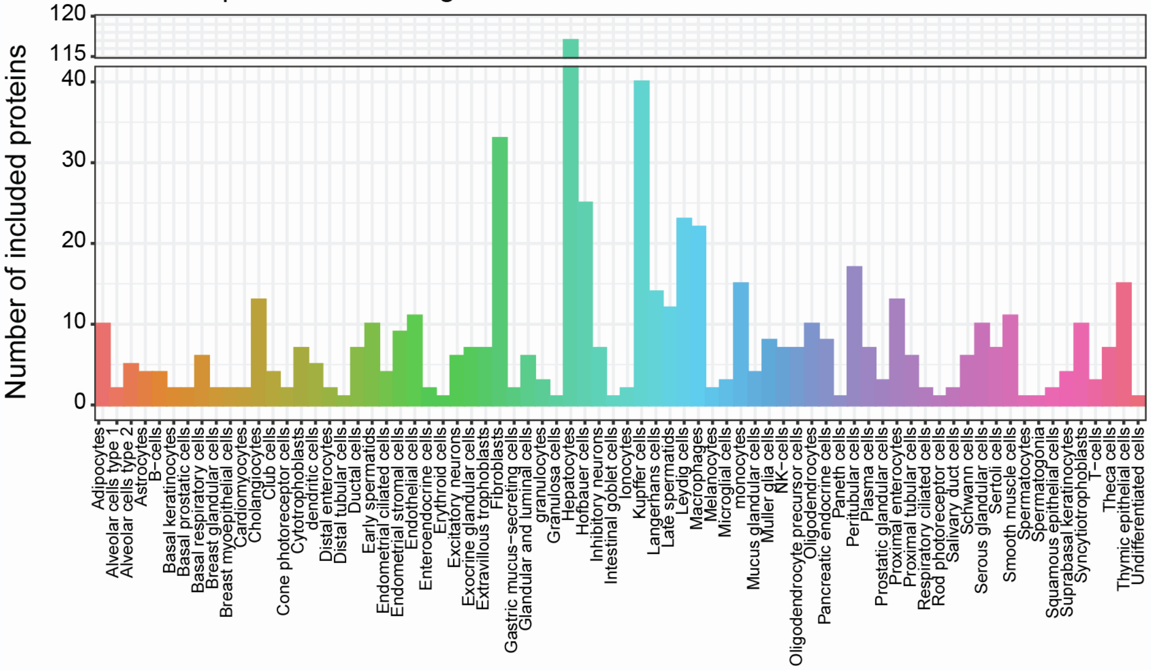

Supplemental Figure S7

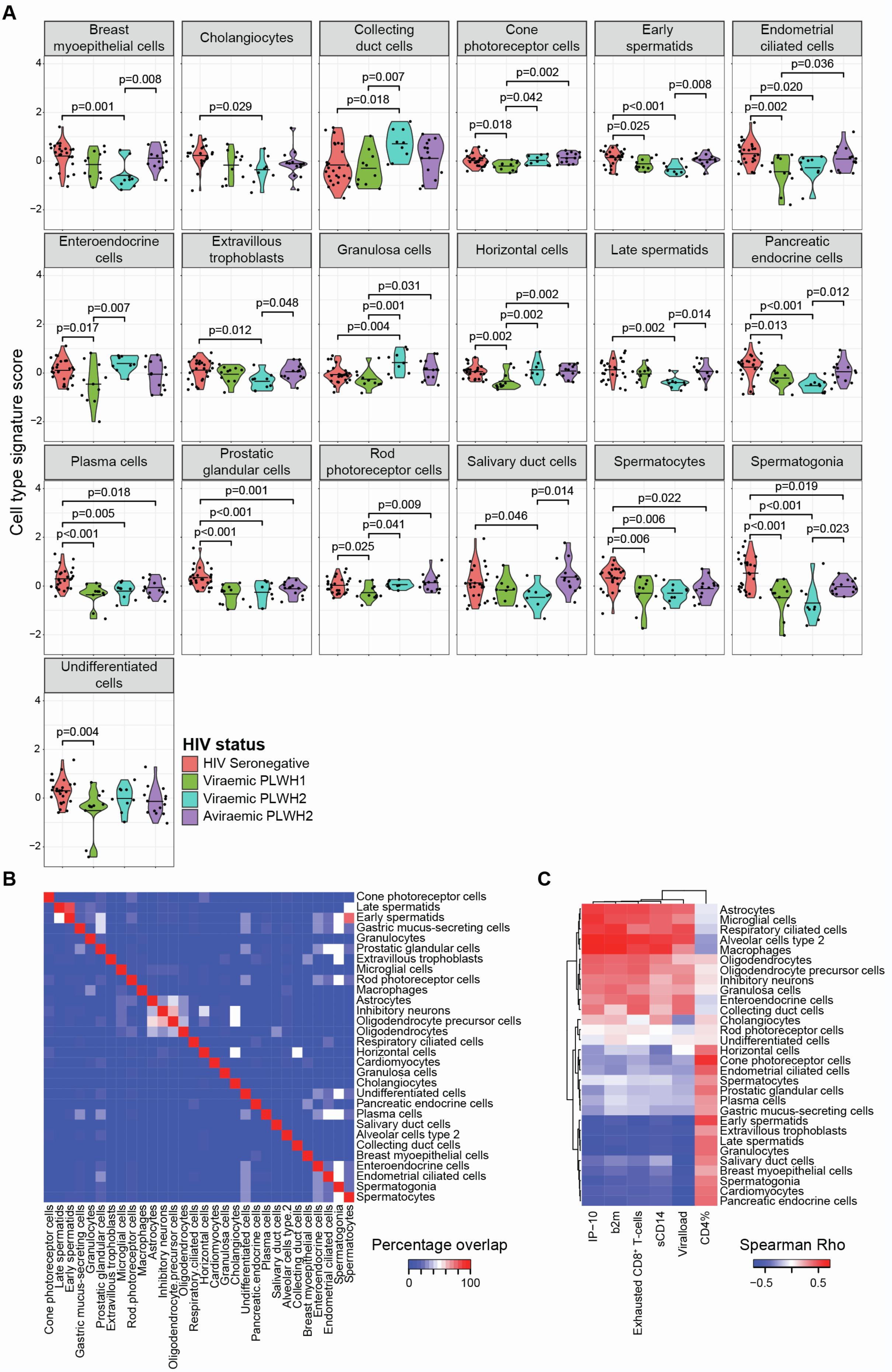

Supplemental Figure S8

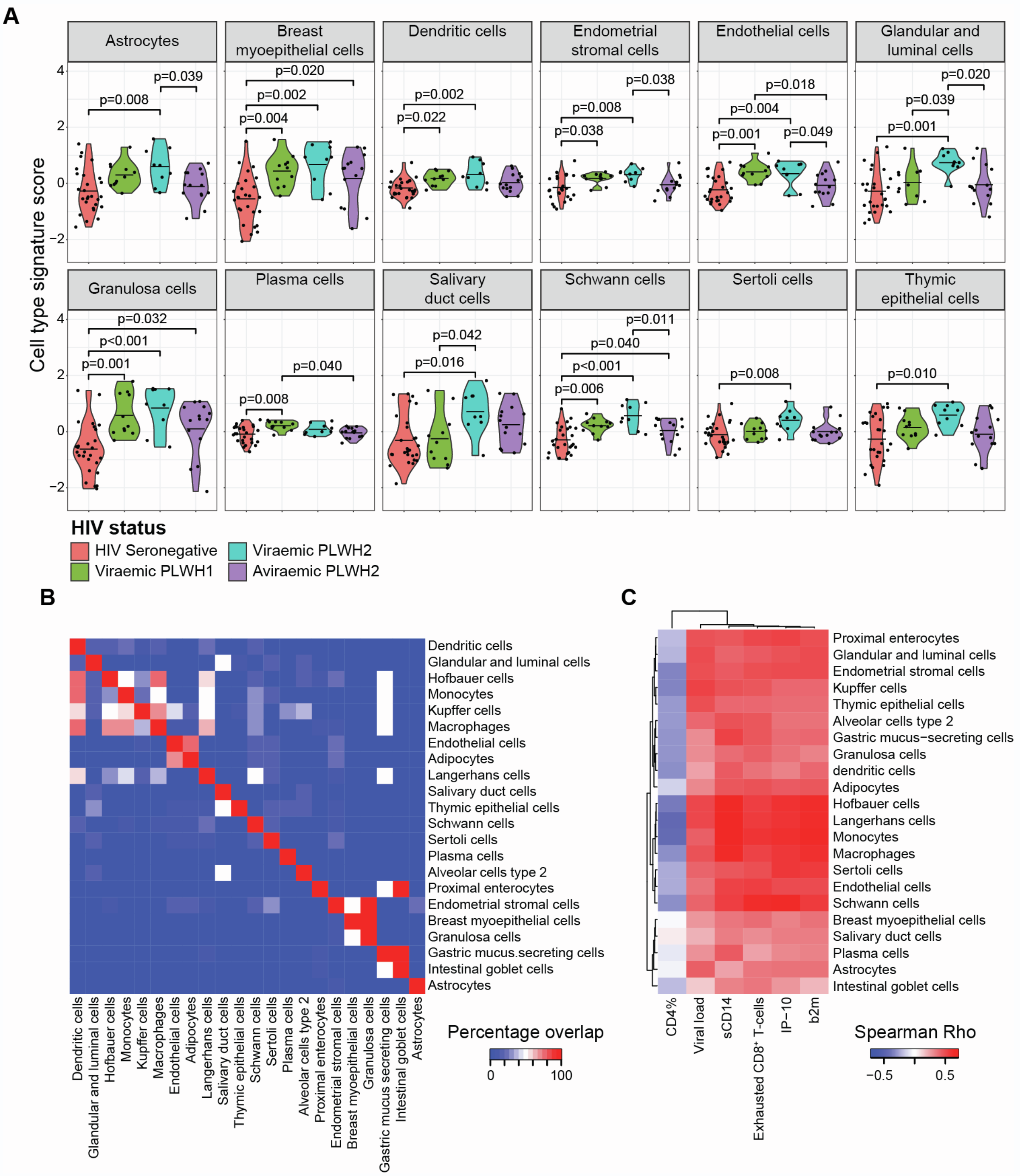

SUPPLEMENTAL FIGURE LEGENDS

Figure S1. Quality control of proteomics data. Related to Figure 1-4. A) Evaluation of eight different normalization methods using the NormalyzedDE package. HIV status groups were used as groups. B) The dimensionality of protein expression was reduced, by performing a principal component analysis (PCA). HIV status, gender, age, sample preparation order, mass-spectrometry running order, and ART treatment status was projected on the PCA plot. C) Plasma proteins differentially expressed in PLWH1 and PLWH2, compared to in HIV seronegative individuals. D) Number of differentially expressed proteins among people living with HIV-1 (PLWH1) (Left circle), HIV-2 (PLWH2) (right circle), and among both PLWH1 and PLWH2 (intersection between two circles). Abbreviations: PCV: mean of 'intragroup coefficients of variation of all replicate groups', PMAD: mean of 'intragroup median absolute deviation across all replicate groups', PEV: mean of 'intragroup pooled estimate of variance across all replicate groups', VSN: variance stabilization normalization; GI: global normalization, CycLoess: cyclic loess normalization, RLR: robust linear regression.

Figure S2. HIV-1 and HIV-2 infections induce similar increases in type I and type II interferon responses. Related to Figure 1. Venn diagrams showing the proportion of differentially expressed protein linked to type I and type II interferon signalling.

Figure S3. Weighted protein co-expression network analysis of plasma proteins. Related to Figure 1. A) Scale-free fit index (left plot) and mean connectivity (right plot) as a function of soft-threshold powers. B) Dotplot displaying the ten biological processes with the highest fold-change in module 1 (left plot) and 2 (right plot). Statistically significant correlations were determined using the Spearman Rank correlation test, followed by Benjamini-Hochberg correction for multiple testing ($p<0.05$).

Figure S3. Top 10 connected hub proteins in weighted protein co-expression network analysis modules 1 and 2. Related to Figure 1. Groupwise comparison of Module 1 (A) and Module 2 (B) top 10 connected hub proteins. Statistically significant group-wise differences were determined by ANOVA test, followed by Benjamini-Hochberg correction for multiple testing ($p<0.05$). Mean values are depicted in violin plots.

Figure S5. HIV infections induces alterations of multiple tissue signatures. Related to Figure 2. A) Bar charts displaying the number of proteins not secreted to blood, i.e. leakage (left plot) and secreted (right plot) protein-derived tissue signatures. B) Spearman correlation and p-value for the correlation between the sigmoid colon (upper plot) and small intestine terminal ileum (lower plot) signatures, and soluble CD14 (sCD14) plasma concentration. Groupwise comparison of C) leakage and D) secreted protein-derived tissue type signatures. Statistically significant correlations were determined using the Spearman Rank correlation test, followed by BH correction for multiple testing ($p<0.05$). Statistically significant group-wise differences were determined by ANOVA test, followed by Benjamini-Hochberg correction for multiple testing ($p<0.05$). Mean values are depicted in violin plots.

Figure S6. Characterisation of cell type signatures. Related to Figure 3 and 4. Bar charts displaying the number of proteins in the leakage (upper plot) and secreted (lower plot) protein-derived cell type signatures.

Figure S7. Both HIV-1 and HIV-2 infections induce alterations of multiple cell type signatures. Related to Figure 3. A) Groupwise comparison of leakage protein-derived cell type signatures. B) Overlap matrix of differentially expressed leakage protein-derived cell type signatures. The frequency represents the percentage of the proteins included in the signature of the cell type in the column, which are included in the signature of the cell type in each row. C) Correlations between cell type signature scores and CD4%, viral load, frequency of exhausted $CD8^+$ T-cells, and plasma concentration of Interferon gamma-induced protein 10 (IP-10, also known as CXCL10), soluble CD14 (sCD14), and b2-microglobulin (b2m), among PLWH2. Statistically significant group-wise differences were determined by ANOVA test, followed by Benjamini-Hochberg (BH) correction for multiple testing ($p<0.05$). Statistically significant correlations were determined using the Spearman Rank correlation test, followed by BH correction for multiple testing ($p<0.05$). Mean values are depicted in violin plots.

Figure S8. HIV infection induces increased secretion of proteins from multiple cell types. Related to Figure 4. A) Groupwise comparison of secreted protein-derived cell type signatures. B) Overlap matrix of differentially expressed secreted protein-derived cell type

signatures. The frequency represents the percentage of the proteins included in the signature of the cell type in the column, which are included in the signature of the cell type in each row. C) Spearman correlation between cell type signature score and CD4%, pVL, frequency of exhausted $CD8^{+}$ T-cells, and plasma concentration of Interferon gamma-induced protein 10 (IP-10, also known as CXCL10), soluble CD14 (sCD14), and b2-microglobulin (b2m), among people living with HIV-2. Statistically significant group-wise differences were determined by ANOVA test, followed by Benjamini-Hochberg (BH) correction for multiple testing ($p<0.05$). Statistically significant correlations were determined using the Spearman correlation test, followed by BH correction for multiple testing ($p<0.05$). Mean values are depicted in violin plots.
